# Neoadjuvant immunotherapy combined with HER2-targeted antibody–drug conjugate in bladder urothelial carcinoma: a case report

**DOI:** 10.1186/s12894-026-02049-w

**Published:** 2026-01-10

**Authors:** Zhengmin Guo, Guodong Wang

**Affiliations:** 1 Department of Pathology, Beijing Electric Power Hospital of State Grid Company of China/Capital Medical University Electric Power Teaching Hospital, Beijing, China; 2 Department of Urology, Beijing Electric Power Hospital of State Grid Company of China/Capital Medical University Electric Power Teaching Hospital, Beijing, China

**Keywords:** Neoadjuvant immunotherapy, Antibody–drug conjugate, Bladder urothelial carcinoma, GC regimen-intolerant

## Abstract

**Background:**

Bladder urothelial carcinoma (UC) represents a prevalent and clinically aggressive malignancy. For cisplatin-intolerant patients, there remains an urgent need to investigate alternative therapeutic regimens. This case report describes the application of combined immunotherapy and HER2-targeted antibody–drug conjugate (ADC) therapy in a patient with gemcitabine plus cisplatin (GC regimen) intolerant bladder UC.

**Case presentation:**

A 66-year-old female patient was diagnosed with human epidermal growth factor receptor 2 (HER2)-positive (immunohistochemistry 2+/3+) bladder UC (T3N0M0). Initial gemcitabine-cisplatin chemotherapy was discontinued due to severe adverse effects. The patient subsequently received four cycles of tislelizumab, an anti-programmed cell death protein-1 monoclonal antibody and five cycles of disitamab vedotin, a HER2-targeted ADC, as neoadjuvant therapy. The treatment was well tolerated, with mild adverse events. Following treatment, the patient underwent laparoscopic radical cystectomy with ileal neobladder reconstruction. The pathological diagnosis revealed treatment-related changes consistent with pathological complete response (pCR), with no recurrence observed during the 9-month postoperative follow-up.

**Conclusion:**

This case demonstrates that neoadjuvant immunotherapy combined with HER2-targeted ADC therapy achieved pCR with a favourable safety profile in a GC regimen-intolerant patient with HER2-positive bladder UC. This result supports the further evaluation of this approach in both clinical studies and real-world practice.

## Background

Bladder cancer represents the tenth most frequently diagnosed malignancy worldwide, with GLOBOCAN 2022 reporting 573,000 new cases and 213,000 deaths annually [1]. Among the histological subtypes of bladder cancer, bladder urothelial carcinoma (UC) is the most prevalent, with muscle-invasive bladder cancer (MIBC), a subtype of muscle-invasive urothelial carcinoma (MIUC), being notably linked to high morbidity and mortality rates [2]. The conventional treatment for MIBC patients typically involves cisplatin-based neoadjuvant chemotherapy (NAC) followed by radical cystectomy [3]. However, some adverse effects, such as nephrotoxicity, ototoxicity and neurotoxicity, preclude the continuation of platinum-based NAC [4, 5].

In response to these limitations, novel therapeutic interventions have emerged, offering promising alternatives. These include immune checkpoint inhibitors (ICIs), FGFR inhibitors, and antibody–drug conjugates (ADCs), which are particularly beneficial for patients who cannot tolerate cisplatin-based regimens [6, 7]. For instance, checkpoint inhibitors like atezolizumab and pembrolizumab have demonstrated enhanced survival benefits in cisplatin-ineligible patients [8]. Furthermore, nivolumab was approved by the FDA for adjuvant therapy of high-risk MIUC, following the Checkmate 274 trial [9]. The HOPE-03 phase Ib/II trial has provided insights into the safety profile and efficacy of the combination of RC-48 and tislelizumab in the neoadjuvant treatment of HER2-positive locally advanced urothelial MIBC [10]. More recently, the RC48-C016 Phase III trial validated the combination of HER2-targeted ADC and immunotherapy as a highly effective and well-tolerated treatment option for cisplatin-ineligible patients, with findings reported in December 2025 [11]. Furthermore, recent data from the phase III KEYNOTE-905/EV-303 trial presented at the ESMO conference held in October 2025, demonstrated that neoadjuvant enfortumab vedotin plus pembrolizumab significantly improved both event-free and overall survival in cisplatin-ineligible MIBC [12].

This case report details an alternative therapeutic approach for a patient with HER2-positive bladder UC who exhibited poor tolerance to gemcitabine plus cisplatin (GC) regimen. The patient achieved a pathological complete response (pCR) following combination therapy with the PD-1 inhibitor tislelizumab and HER2-targeted ADC disitamab vedotin, providing evidence for this emerging regimen.

## Case presentation

**Fig. 1:**
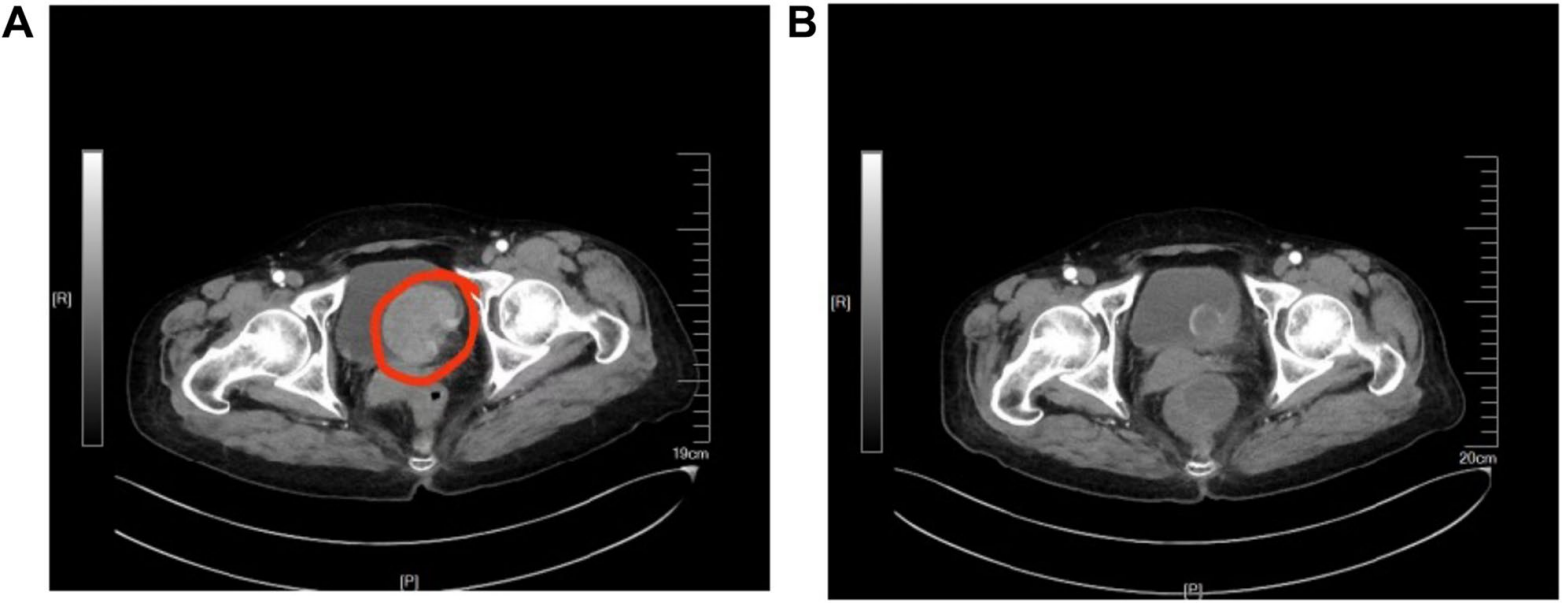
Contrast-enhanced CT imaging of the bladder before and after neoadjuvant therapy. **A **Baseline imaging: Axial CT image performed on November 29, 2024, prior to the initiation of neoadjuvant therapy, demonstrated a large, irregular mass (indicated by “O”) arising from the left posterior bladder wall. **B** Post-treatment imaging: Axial CT image performed on March 10, 2025, following completion of neoadjuvant therapy with tislelizumab and disitamab vedotin, showed significant regression of the previously noted mass, with only minimal residual soft tissue thickening or scarring at the primary tumour site

A 66-year-old female patient presented to the outpatient clinic on November 23, 2024, with a two-week history of urinary frequency, urgency and dysuria, accompanied by gross haematuria for the past two days. She was evaluated and admitted on the same day. Two weeks before admission, the patient developed symptoms of urinary irritation without any identifiable precipitating factors. There was no associated fever, flank pain, abdominal discomfort or other accompanying symptoms. Two days prior to admission, she experienced haematuria with visible blood clots. Based on the clinical presentation, a preliminary diagnosis of urinary tract infection and haematuria was made, and the patient was admitted for further evaluation. Renal ultrasonography showed bilateral renal atrophy, with the right kidney measuring 8.3 cm × 3.3 cm and the left kidney 9.3 cm × 3.3 cm. A fixed, solid isoechoic mass approximately 4.9 cm × 3.8 cm in size was identified on the left lateral wall of the bladder, with detectable internal blood flow on Doppler imaging. Computed tomography urography (CTU) revealed a pedunculated mass measuring approximately 53 mm × 42 mm on the left posterior wall of the bladder (Fig. 1A), exhibiting heterogeneous enhancement on both noncontrast and contrast-enhanced scans. The lesion had an irregular serosal surface and indistinct surrounding fat planes. Imaging findings were strongly suggestive of bladder carcinoma with possible extravesical invasion.

### Diagnosis

**Fig. 2:**
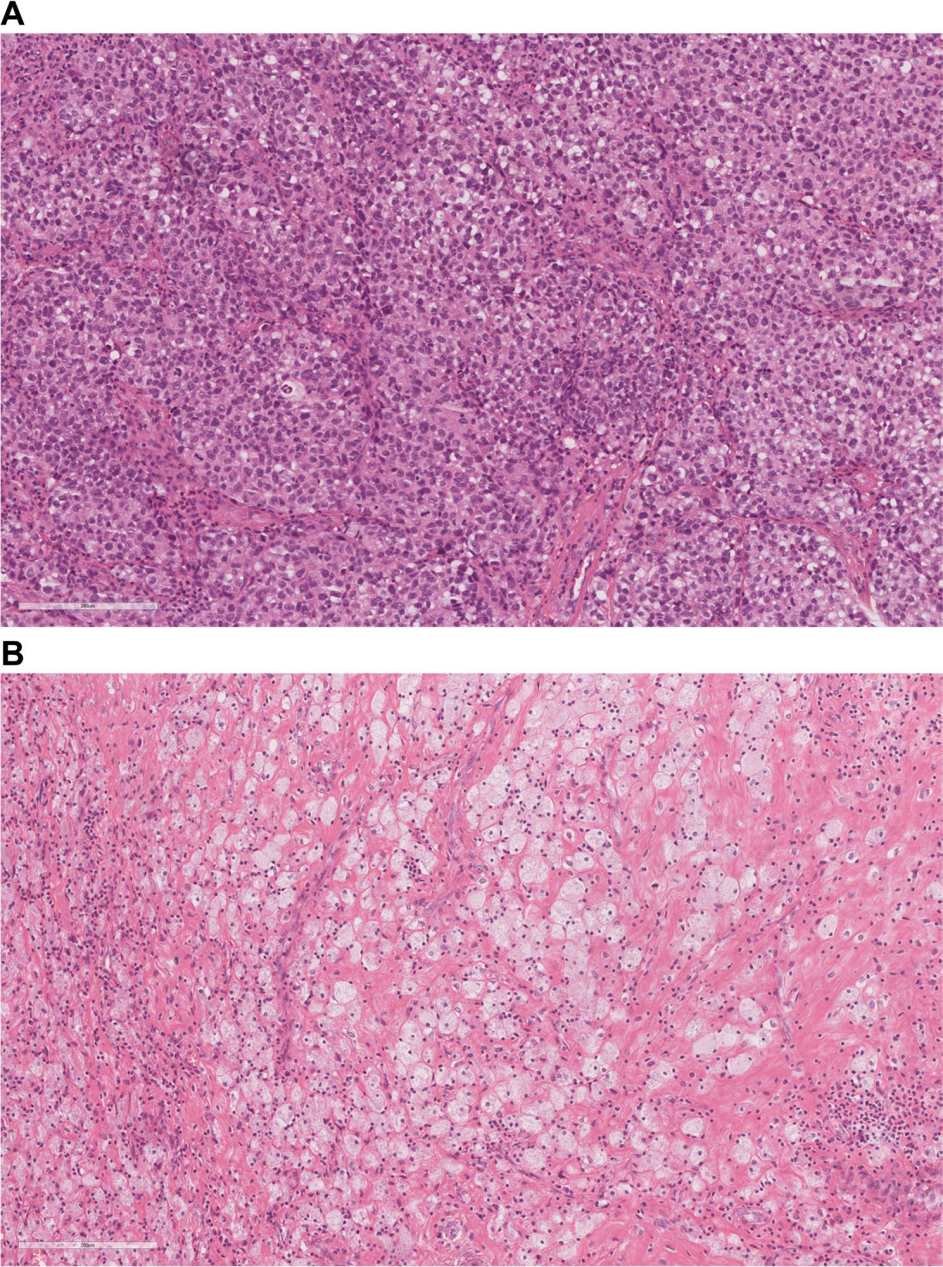
Histopathological comparison of bladder tissue before and after neoadjuvant therapy (haematoxylin and eosin staining, 200 × magnification). **A** Pre-treatment biopsy (December 10, 2024): Typical morphology of high-grade urothelial carcinoma, characterised by disorganised architecture, pleomorphic nuclei and frequent mitotic figures. **B **Post-treatment cystectomy specimen (March 12, 2025): No viable tumour cells were identified. The tumour bed was largely replaced by extensive foamy macrophage infiltration and dense collagenous fibrosis, consistent with pathological complete response to treatment The scale line at the lower left corner of the picture represents 200 μm

On December 9, 2024, the patient underwent transurethral resection (a diagnostic partial resection) of the bladder tumour. Specifically, three targeted samples were obtained from the tumor mass during the procedure to confirm histopathological diagnosis and assess tumor characteristics. Postoperative histopathological analysis confirmed a diagnosis of high-grade bladder UC (Fig. 2A). Immunohistochemical staining revealed the following profile: CK7 (positive), CK20 (positive), CK34βE12 (focally positive), P63 (positive), GATA-3 (positive), Ki-67 (approximately 80% positive), Chromogranin A (CgA, negative), Synaptophysin (Syn, negative), S-100 (negative), PAX-8 (negative) and Vimentin (negative). Based on clinical, imaging and pathological findings, the patient was diagnosed with bladder UC, which was staged as T3N0M0.

**Fig. 3:**
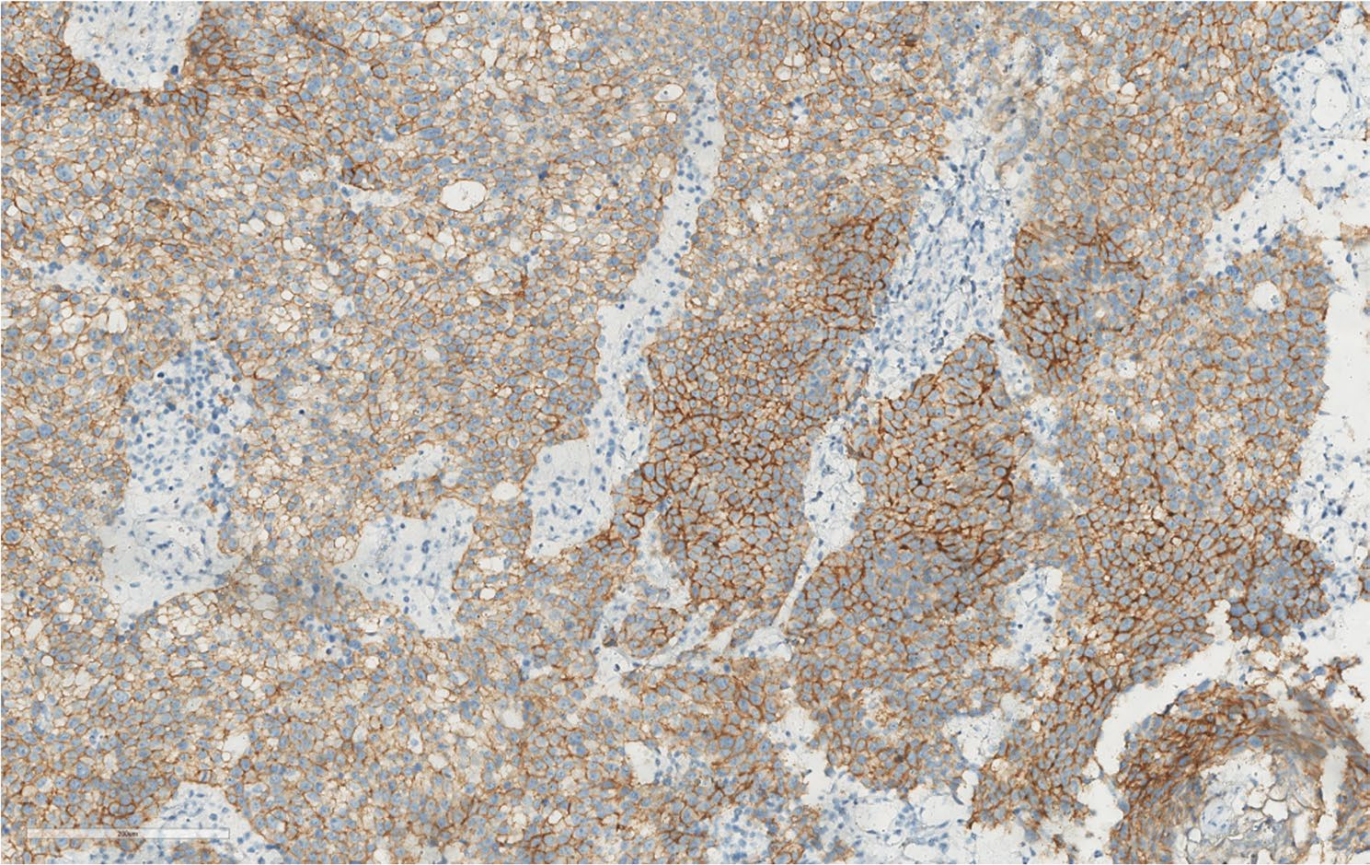
Immunohistochemical (IHC) staining for human epidermal growth factor receptor 2 (HER2) expression of the bladder tumour. The specimen exhibited heterogeneous HER2 protein expression (2 + with focal areas of 3 + intensity) (200 × magnification). The scale line at the lower left corner of the picture represents 200 μm

The initial treatment plan consisted of neoadjuvant chemotherapy with gemcitabine plus cisplatin (GC regimen) in combination with immunotherapy using tislelizumab. However, on December 20, 2024, the patient received the first intravenous infusion of gemcitabine (1.75 g), after which she developed severe adverse effects. The patient experienced severe toxicities including nausea, vomiting, headache and dizziness following the first dose of gemcitabine, which were highly distressing and prompted discontinuation of the entire GC regimen. The marked intolerance to gemcitabine strongly suggested that the patient would poorly tolerate subsequent cisplatin as well. Given the anticipated higher toxicity profile of cisplatin, the patient was deemed intolerant to platinum-based chemotherapy. The immunohistochemistry analysis of the bladder tumour confirmed human epidermal growth factor receptor 2 (HER2) positivity (2+, focal area 3+) (Fig. 3). According to the disitamab vedotin prescribing information, this agent is indicated for patients with HER2-overexpressing (IHC 2 + or 3+) who have previously received platinum-containing chemotherapy. Consequently, the treatment regimen was modified to immunotherapy with tislelizumab (200 mg, intravenous infusion, once every 3 weeks) in combination with the anti-HER2 ADC-disitamab vedotin (120 mg, intravenous infusion, once every 2 weeks). The dosing regimen was based on the instructions for the use of PD-1 and the relevant content of the Phase 3 RC48-016 clinical trial (NCT05302284), whose trial protocol was published in 2023. Between December 23, 2024, and February 28, 2025, the patient received four cycles of tislelizumab and five cycles of disitamab vedotin. Following completion of neoadjuvant therapy with tislelizumab and disitamab vedotin, CTU showed significant regression of the previously noted mass (Fig. 1B). The main adverse events during treatment were mild nausea, decreased appetite and numbness in the fingertips, all of which were generally well tolerated. The adverse events were reported based on the patient’s description and were not retrospectively graded.

**Fig. 4:**
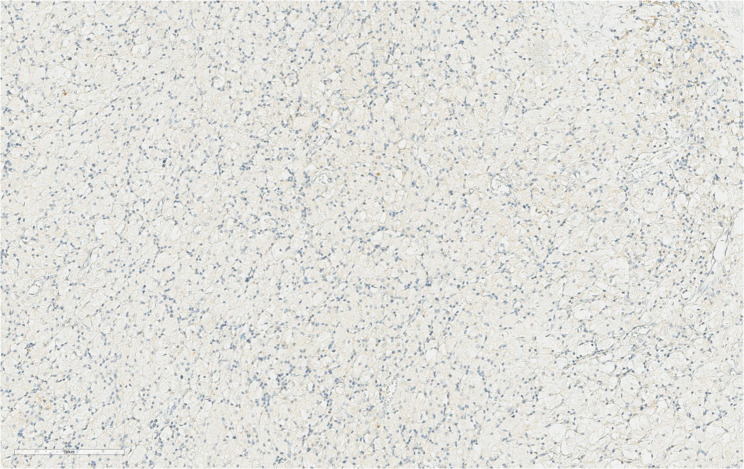
Immunohistochemical (IHC) staining for CK marker (cytokeratin) of the bladder tumour. Post-treatment (March 11, 2025), the expression of CK marker by immunohistochemistry was negative (200× magnification). The scale line at the lower left corner of the picture represents 200 μm

On March 11, 2025, the patient underwent laparoscopic radical cystectomy (female type) with ileal neobladder reconstruction. Gross examination of the resected bladder revealed a grey-red, broad-based, spherical mass measuring 3.5 cm × 2.5 cm × 2.0 cm at the site of the original tumour, with a gelatinous cut surface. Histological examination of the entire tumour bed revealed no residual malignant cells. Microscopically, there was a dense infiltration of foamy histiocytes accompanied by stromal fibrosis with collagen deposition and foci of calcification. The pathological diagnosis indicated treatment-related changes, consistent with pCR (Fig. 2B). The post-treatment immunohistochemical analysis revealed absence of cytokeratin (CK) expression (Fig. 4). This result aligns with the patient’s pathological pCR and provides additional objective evidence of treatment efficacy. At approximately 9 months postoperatively, the patient remained clinically stable, with no signs of recurrence or complications. Recovery was uneventful, and the fingertip numbness experienced during neoadjuvant therapy had improved. No immune-related adverse events (irAEs) were observed during the treatment period and the follow-up period. All monitored immune-related parameters (cardiac enzymes, thyroid function, complement levels, and inflammatory markers) were within normal ranges. Given the patient’s initial clinical stage (≥ T3) and subsequent pCR status, we have implemented a surveillance protocol consisting of quarterly evaluations (including abdominopelvic CT imaging, renal function tests, and serum biomarker analysis) for the first three postoperative years, followed by annual assessments thereafter.

## Discussion and conclusions

There remains a significant unmet need for effective alternative therapies in cisplatin-ineligible patients with MIBC. This challenge is underscored by a previously reported case of bladder UC in which the patient underwent laparoscopic surgery and cisplatin-gemcitabine chemotherapy but later experienced severe treatment-related adverse effects, leading to therapy discontinuation and subsequent death from acute cerebral infarction 3 months post-surgery [13]. The present case involved a bladder UC (T3N0M0) patient who experienced severe gemcitabine-associated toxicity during planned cisplatin-based NAC, prompting a change in the treatment strategy. Given the HER2-positive status (2+/focal 3+), the regimen was transitioned to a combination therapy with the PD-1 inhibitor tislelizumab and HER2-targeted ADC disitamab vedotin, ultimately achieving pCR.

The combination of ADC with immunotherapy demonstrates significant potential for synergistic effects. Not only directly killing tumor cells through targeted delivery of cytotoxic drugs, but also inducing immunogenic cell death (ICD) and enhancing immune responses in the tumor microenvironment [14]. The rationale for combining ICIs with ADCs lies in potential synergistic mechanisms. ADCs can induce ICD and enhance antigen presentation, potentially augmenting the efficacy of ICIs [14]. Additionally, targeting HER2 with disitamab vedotin may enhance immune activation in HER2-positive UC. Although limited data exist on this combination in neoadjuvant MIBC, emerging studies in metastatic settings have indicated promising efficacy and a manageable safety profile [15, 16]. A recent multicenter phase Ib/II study, HOPE-03, evaluated disitamab vedotin (RC48-ADC) combined with tislelizumab as neoadjuvant therapy for HER2-positive, locally advanced MIBC, showing encouraging safety and efficacy results, which supports the therapeutic approach used in our case [10]. Moreover, studies such as the one by Sheng et al. evaluating disitamab vedotin plus toripalimab in HER2-expressing advanced UC, and the RC48-C014 trial evaluating disitamab vedotin plus toripalimab in patients with locally advanced or metastatic UC, also provide supportive evidence for the efficacy of combining HER2-targeted ADCs with ICIs [11, 17]. The integration of immunotherapy (tislelizumab, an anti-PD-1 antibody) and disitamab vedotin (an ADC targeting HER2) in the neoadjuvant setting yielded an excellent response—confirmed by the absence of residual carcinoma and prominent treatment-related changes (foam cell infiltration, fibrosis, calcification)—and, ultimately, pCR at radical cystectomy. The regimen was well tolerated, with only mild nausea, anorexia and reversible fingertip numbness [16].

Clinically, achieving pCR is associated with improved long-term outcomes in MIBC, including overall survival and disease-free survival. Recent real-world studies also support the efficacy of disitamab vedotin combined with immunotherapy in cisplatin-ineligible MIBC patients [18, 19]. Our case revealed pCR with the tislelizumab and disitamab vedotin combination, suggesting a viable and effective alternative for platinum-unfit bladder UC patients, consistent with growing real-world observations. The underlying pathophysiological mechanisms, particularly the role of HER2 2 + status in enhancing immune activation, warrant further investigation. Additionally, the recent approval of trastuzumab deruxtecan, another HER2-targeted ADC for metastatic UC with high HER2 expression (HER2 3+), highlights the evolving landscape of HER2-targeted therapies in UC [20, 21].

To our knowledge, this case represents the first reported case combining a PD-1 inhibitor with disitamab vedotin (an HER2-targeting ADC) in a GC regimen-intolerant bladder UC patient. The limitations of this case report include the inherent lack of generalisability and the relatively short follow-up period. While postoperative surveillance at 9 months revealed no recurrence or major complications, the short follow-up period prevents definitive assessment of long-term outcomes. Finally, the optimal dosing regimen and treatment schedule for this combination therapy need to be validated through prospective clinical studies.

In summary, this case demonstrates the clinical potential of neoadjuvant immunotherapy combined with ADC for GC regimen-intolerant bladder UC. The achievement of pCR, coupled with a manageable toxicity profile, provides a compelling rationale for the further evaluation of this approach in both clinical studies and real-world practice. Future research should focus on protocol optimisation and the comprehensive assessment of long-term survival benefits.

## Data Availability

The datasets used and/or analysed in the study are available from the corresponding author upon reasonable request.

## Abbreviations

UC: Urothelial carcinoma
ADC: Antibody–drug conjugate
HER2: Human epidermal growth factor receptor 2
MIBC: Muscle-invasive bladder cancer
pCR: Pathological complete response
CTU: Computed tomography urography
ICD: Immunogenic cell death
